# Can people afford to pay for health care? Evidence on inequity in financial protection in Europe

**DOI:** 10.1093/eurpub/ckac128.001

**Published:** 2022-10-25

**Authors:** Organised by: WHO Europe, Moderator: Natasha Azzopardi Muscat (WHO Europe)

## Abstract

Governments have repeatedly affirmed their commitment to meeting the goals of universal health coverage (UHC) – to ensure that everyone can use the quality health services they need without experiencing financial hardship. In spite of strong political commitment to UHC, research from the WHO Regional Office for Europe shows that:

- gaps in health coverage lead to unmet need for health care and financial hardship among people using health services
- these negative outcomes are heavily concentrated among people in poverty and those with multiple chronic conditions
- countries can reduce unmet need and financial hardship by re-designing coverage policy (the way in which health coverage is designed and implemented)

This session aims to raise awareness about the most prevalent gaps in coverage in European health systems, the policies that cause them and what countries can do to address them. It will draw on findings from an updated version of the WHO study ‘Can people afford to pay for health care? New evidence on financial protection in Europe’, which covers over 35 countries in Europe, including all EU member states and many middle-income countries

The session will highlight common gaps in coverage that systematically harm people with low incomes:

- the basis for population entitlement to publicly financed health services: exclusion of undocumented migrants; linking entitlement to employment or payment of contributions
- the scope and quality of publicly financed health services: poor coverage of medicines and dental care for adults; unreasonable waiting times leading to use of private services or non-covered medicines and unmet need
- user charges (co-payments): the absence of exemptions for people with low incomes; the absence of annual caps on all user charges; heavy reliance on percentage co-payments

The session will focus on how to make progress by:

- drawing attention to key principles for re-designing coverage policy, especially for people who are ‘left behind’ (adopting progressive universalism)
- showing how countries in Europe have done this, using examples of good practice

**Keynote speaker:**

**Charles Normand**

Trinity College Dublin, Dublin, Ireland

**Panellists:**

**Sarah Thomson**

WHO Europe

**Kaisa Immonen**

European Patients' Forum (EPF)

